# Inferring Multiple Refugia and Phylogeographical Patterns in *Pinus massoniana* Based on Nucleotide Sequence Variation and DNA Fingerprinting

**DOI:** 10.1371/journal.pone.0043717

**Published:** 2012-08-29

**Authors:** Xue-Jun Ge, Tsai-Wen Hsu, Kuo-Hsiang Hung, Chung-Jian Lin, Chi-Chung Huang, Chao-Ching Huang, Yu-Chung Chiang, Tzen-Yuh Chiang

**Affiliations:** 1 Key Laboratory of Plant Resources Conservation and Sustainable Utilization, South China Botanical Garden, Chinese Academy of Sciences, Guangzhou, China; 2 Endemic Species Research Institute, Nantou, Taiwan; 3 Graduate Institute of Bioresources, Pingtung University of Science and Technology, Pingtung, Taiwan; 4 Department of Life Sciences, Cheng Kung University, Tainan, Taiwan; 5 Department of Biological Sciences, National Sun Yat-sen University, Kaohsiung, Taiwan; University of Oxford, United Kingdom

## Abstract

**Background:**

*Pinus massoniana*, an ecologically and economically important conifer, is widespread across central and southern mainland China and Taiwan. In this study, we tested the central–marginal paradigm that predicts that the marginal populations tend to be less polymorphic than the central ones in their genetic composition, and examined a founders' effect in the island population.

**Methodology/Principal Findings:**

We examined the phylogeography and population structuring of the *P. massoniana* based on nucleotide sequences of cpDNA *atpB-rbcL* intergenic spacer, intron regions of the *AdhC2* locus, and microsatellite fingerprints. SAMOVA analysis of nucleotide sequences indicated that most genetic variants resided among geographical regions. High levels of genetic diversity in the marginal populations in the south region, a pattern seemingly contradicting the central–marginal paradigm, and the fixation of private haplotypes in most populations indicate that multiple refugia may have existed over the glacial maxima. STRUCTURE analyses on microsatellites revealed that genetic structure of mainland populations was mediated with recent genetic exchanges mostly via pollen flow, and that the genetic composition in east region was intermixed between south and west regions, a pattern likely shaped by gene introgression and maintenance of ancestral polymorphisms. As expected, the small island population in Taiwan was genetically differentiated from mainland populations.

**Conclusions/Significance:**

The marginal populations in south region possessed divergent gene pools, suggesting that the past glaciations might have low impacts on these populations at low latitudes. Estimates of ancestral population sizes interestingly reflect a recent expansion in mainland from a rather smaller population, a pattern that seemingly agrees with the pollen record.

## Introduction

Climate changes over the past two million years are often interpreted as the primary driver of range fragmentation and the speciation of animals and plants in the northern hemisphere [1]. Facing the crisis of extinction, plant species often responded through their dispersal to regions with environmental conditions where they could survive over the glacial cycles [2]. Footprints of demographic histories that the species evolved through were retained in the individuals' genomes. Phylogeography, the study of the spatio-temporal dynamics of populations, relies on inferences from macrofossils and pollen in sediment profiles and molecular evidence that can also reveal historical aspects such as the location of cryptic refugia, especially when palaeoecological evidence is not available. From the molecular evidence, the number of genetically distinct, ancestral lineages, their locations during the glacial maxima, and the postglacial migration routes for tree and plant species could be inferred [3], [4].

Because a species is spatially structured into populations, the population size and extent of interpopulation gene flow would determine the levels of genetic diversity within populations/species. A central–marginal paradigm predicts that populations around the distributional center tend to possess higher levels of genetic variation than the peripheral ones because genetic variations would be lost through stochastic drift faster in marginal populations than in the central ones [5]. Furthermore, islands are natural laboratories for studying plant evolution, and have long been attractive to evolutionary biologists. Once an island with small geographical size is colonized, population differentiation between islands, as well as between island and neighboring mainland, would be facilitated by founders' effects and local extinction-recolonization [6]. Compared to the volcanic origin of oceanic islands, continental islands emerge almost simultaneously via collision between continent and oceanic tectonic plates. In Taiwan, most species/populations thus have close, phylogenetic links to their mainland relatives. Species like *Pinus luchuensis* and *P. taiwanensis* [7], *Cycas taitungensis* and *C. revoluta* [8] indicate a pattern of colonization from the Asian continent eastward to Taiwan. Compared to ancestral populations on the mainland, one would expect island populations maintains lower genetic variability as the result of smaller population sizes and numbers due to habitat limitation on islands as well as genetic bottlenecks associated with colonization.

*Pinus massoniana* Lamb., a species of sect. *Pinus*, is distributed in central and southern mainland China and Taiwan [9]. The species is ecologically dominant on the mainland, whereas has a single population in Taiwan that remains small with fewer than 100 individuals in the wild, although locally dominant. As a light-demanding and long-lived tree, *P. massoniana* acts as an ecological pioneer, colonizing mesic, harsh sites and competing little with other woody plants [10], [11]. Such attributes are valuable for revegetation after logging or mining [12], especially as China vows to improve forest management and to increase wood production in order to meet the escalating domestic demands and to reduce the smuggling of logs [13]. In South China, timber plantation is predominantly conducted by growing *Cunninghamia lanceolata* [14] and *Pinus massoniana* [9] due to their fast growth or delicate wood texture.

As a common species, mainland populations are likely mediated by some degree of gene flow, especially via wind-dispersed pollen [10]. Nevertheless, vicariance events, such as formation of the Wu-yi mountain range in China, would counteract the homogeneous force of gene flow from the isolation of the population fragments. Furthermore, gene flow between Taiwan and the geographically near populations, such as those in Fujian and Guangdong provinces, can be hindered because of the barrier from the Taiwan Strait. With no or few immigrants, random genetic drift would likely drive losses of genetic variations from the small-sized population. Low levels of genetic diversity within the peripheral population and genetic distinctness from mainland populations would therefore be expected in the Taiwan population [8]. Altogether, phylogeography and genetic characteristics of populations/species are dictated by the interplay between historical vicariance and recurrent genetic exchanges [15]. These evolutionary events would leave evolutionary footprints in the spatial apportionment of genetic variations within and between populations across the distributional range of *P. massoniana*, enabling one to recover the demographic histories [16]. Of the geological events, historical glacial cycles on the Eurasia Continent had prevalent influences on the survival (extinction) and recolonization of populations/species. According to the geological evidence, during the early Pleistocene, ice ages occurred at regular intervals of 100000 years followed by a 20000-year warm period (Milankovitch cycles) [17]. Like many other gymnosperms and angiosperms in East Asia, *P. massoniana* also survived glacial cycles [15]. Pollen data suggest that southeastern China was forested throughout the Quaternary [18], and *Pinus* has generally been an important component of the regional vegetation. Many Chinese subtropical *Pinus* dominated the vegetation during the late Pleistocene and Holocene with only minor population changes [19], [20].

The distribution of a species across mainland and island provides a perfect system for testing the central–marginal paradigm [5]. In the study, we examined the genetic variation and structure across the natural populations of *P. massoniana* based on sequence variation in the *atpB-rbcL* intergenic spacer of cpDNA, the introns 4 to 8 of *AdhC2* gene of nuclear DNA, and 11 microsatellite loci. Both nuclear and cpDNA markers can be dispersed via pollen and seed, as well as nuclear microsatellite loci which are biparentally inherited. The cpDNA of *Pinus* is exclusively carried by pollen, a pattern unlike most other plants. *AdhC* is one member of the *Adh* gene family, which encodes a key enzyme, alcohol dehydrogenase, in the glycolytic pathway. Fast molecular evolution and near neutrality make its intron regions suitable for population study [21], [22], [23], [24]. The following phylogeographical questions were examined: (1) Considering the central–marginal paradigm [5], were the marginal *Pinus* populations less polymorphic than the central ones in their genetic composition? (2) Did the marginal populations in south region act some refugia over the glacial maxima? And (3) how and when was the island population colonized by migrants from the adjacent mainland? Has the Taiwan Strait acted as a barrier and caused genetic differentiation between the populations of mainland and Taiwan, especially given effects of founders' effects in the latter?

## Results

### Sequence variation and genetic diversity

**Figure 1:**
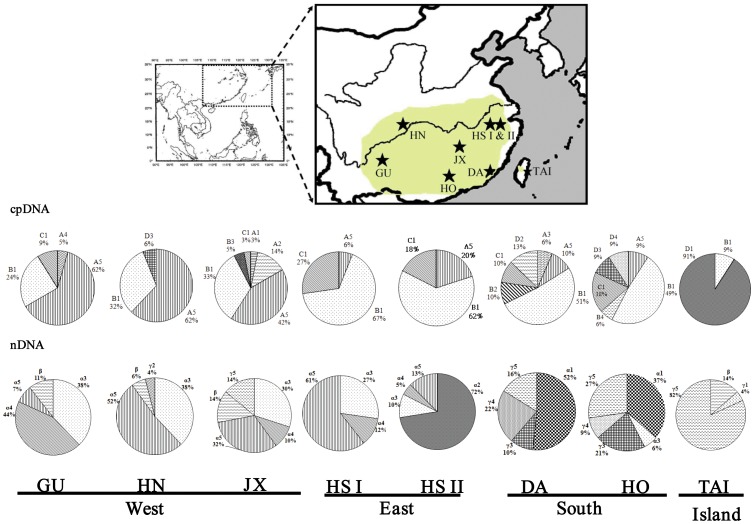
Map showing the distribution and genetic composition of populations of *Pinus massoniana* in mainland China and Taiwan (Pale-green region: species range). Frequencies of cpDNA and nDNA haplotypes in each population are indicated by pie diagrams. Abbreviations of populations are given in Table 1.

We examined the genetic diversity of cpDNA and nuclear DNA within and between populations of *Pinus massoniana*. Eight populations of *Pinus massoniana* throughout its whole geographical range were surveyed (Fig. 1, Table 1). All haplotype sequences were deposited in the GenBank database (accession numbers of cpDNA: HE971712-HE971725; accession numbers of nDNA: HE972218-HE972268). In total, 24 and 39 polymorphic sites, with 0, 10 and 6 recombinations, were detected in the *atpB-rbcL* intergenic spacer, and *AdhC2* introns, respectively.

**Table 1:** Locations, codes and sample sizes of sampled populations in Taiwan, and geographical populations (East, West, and South) of mainland China of *Pinus massoniana*.

Regions	Locations	Coordinates	Code	Sample size
**Mainland China:**				
**East**				
	Huangshan I, Anhui	30°07′N, 118°11′E	HS I	33
	Huangshan II, Anhui	30°07′N, 118°11′E	HS II	40
**West**				
	Lushan, Jiangxi	29°33′N, 115°59′E	JX	66
	Zhangjiajie, Hunan	29°20′N, 110°25′E	HN	50
	Fanjingshan, Guizhou	27°54′N, 108°39′E	GU	45
**South**				
	Daiyunshan, Fujian	25°39′N, 118°13′E	DA	31
	Yangshan, Guangdong	24°49′N, 112°07′E	HO	33
**Island Taiwan:**				
	Miaoli, Taiwan	24°29′N, 120°72′E	TAI	22

**Figure 2:**
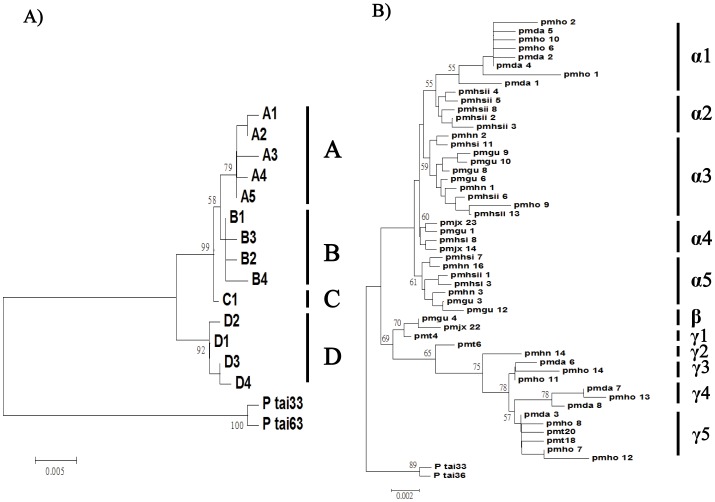
The maximum likelihood tree of *Pinus massoniana* with at outgroup sequences. Divergence times between major lineages and bootstrap values are indicated at nodes. A) cpDNA haplotypes; B) alleles of *AdhC2* introns.

Fourteen cpDNA haplotypes of (A1–A5, B1–B4, C1 and D1–D4) were identified (Fig. 2A, Table 2). Of these, only four haplotypes (A5, B1, C1, and D3) were shared between populations; while 10 others were private to single populations. Among the chlorotypes, haplotype B1 was most frequent (40.6%), followed by haplotype A5 (32.2%). Populations Daiyunshan of Fujian (DA) and Yangshan of Guangdong (HO) in south region possessed the highest level of nucleotide diversity, θ = 0.00696 and 0.00672, respectively; while two populations of Huangshan possessed the lowest level of genetic diversity, θ = 0.00090 and 0.00085, respectively (Table 3).

**Table 2:** Distribution of cpDNA haplotypes in Taiwan population, and geographical populations (East, West, and South) of mainland China *Pinus massoniana.*

Region	Population			A				B			C		D			
		A1	A2	A3	A4	A5	B1	B2	B3	B4	C1	D1	D2	D3	D4	No. of cytotypes
Interior vs. Exterior nodes		E	I	E	E	I	I	E	E	E	I	I	E	I	E	(no. of private types)
**Mainland China:**																
**East**																
	HS I					2	22				9					3 (0)
	HS II					8	25				7					3 (0)
**West**																
	JX	2	9			28	22		3		2					6 (3)
	HN					31	16							3		3 (0)
	GU				2	28	11				4					4 (1)
**South**																
	DA			2		3	16	3			3		4			6 (3)
	HO					3	16			2	6			3	3	6 (2)
**Island Taiwan:**																
	TAI						2					20				2 (1)
Total		2	9	2	2	103	130	3	3	2	31	20	4	6	3	

**Table 3:** The haplotype numbers (Hap.), nucleotide diversity (θ), and neutrality tests at the *atpB-rbcL* spacer of cpDNA and the intron of *AdhC2* of nDNA within Taiwan population, and geographical populations (East, West, and South) of mainland China of *Pinus massoniana*.

Mainland China:	Hap.		θ		Fu and Li's *D*		Fu and Li's *F*		Tajima's *D*	
	cpDNA	nDNA	cpDNA	nDNA	cpDNA	nDNA	cpDNA	nDNA	cpDNA	nDNA
East										
HS I	3	8	0.00090	0.00266	−0.33034	1.27814	−0.35947	1.40390	−0.28956	1.14714
HS II	3	9	0.00085	0.00302	0.95275	0.67857	0.83322	0.60882	0.09664	0.12704
West										
JX	6	9	0.00216	0.00317	−1.63654	−0.51291	−1.64220	−0.35815	−0.87383	0.21384
HN	3	7	0.00514	0.00517	−2.76648**	−0.59590	−2.90467**	−0.82140	−1.88781*	−1.02384
GU	4	9	0.00121	0.00564	−1.08108	−0.05394	−1.08324	−0.01401	−0.57961	0.09712
South										
DA	6	8	0.00696	0.01205	−1.83464	0.66778	−2.03715	0.89330	−1.72970	1.20269
HO	6	12	0.00672	0.01558	0.70711	−0.76809	0.48060	−0.70068	−0.40404	−0.18716
**Island Taiwan:**										
TAI	2	5	0.00488	0.00396	1.48091*	−1.71912	0.84973	−2.03545	−1.12149	−1.85502*
**Overall**	14	51	0.00596	0.01142	−1.77173	−4.01321**	−1.24552	−3.18093**	0.12439	−0.66249

In total, 51 alleles of the *AdhC2* introns were identified. They were clustered into three types, α, β, and γ (Fig. 2B, Table 4). Clusters α1, γ3, and γ4 were restricted to populations in the south region; while α2 were private to populations of Huangshan (east region) (Table 4). The nucleotide diversity (θ) of all populations as a whole was 0.01142. Population Yangshan (HO) in south region had the highest level of genetic diversity, θ = 0.01558, followed by Daiyunshan (DA) (θ = 0.01205); while populations of Huangshan had the lowest levels of genetic diversity, θ = 0.002666 and 0.00302 in mainland China (Table 3).

**Table 4:** Distribution of nucleotypes in Taiwan population, and geographical populations (East, West, and South) of mainland China of *Pinus massoniana*.

Region	Population			α			β			γ			
		α1	α2	α3	α4	α5	β	γ1	γ2	γ3	γ4	γ5	No. of cytotypes
Interior vs. Exterior nodes		E	I	I	E	I	I	I	E	E	E	E	(no. of private types)
**Mainland China:**													
**East**													
	HS I			9	4	20							4 (0)
	HS II		29	4	2	5							5 (1)
**West**													
	JX			25	29	5	7						4 (0)
	HN			19		26	3		2				4 (1)
	GU			15	5	16	7					2	5 (0)
**South**													
	DA	16								3	7	5	4 (0)
	HO	12		2						7	3	9	5 (0)
**Island Taiwan:**													
	TAI						3	1				18	3 (1)
Total		28	29	74	40	72	20	1	2	10	10	34	

### Gene genealogy and nested cladistic analysis of DNA haplotypes

**Figure 3:**
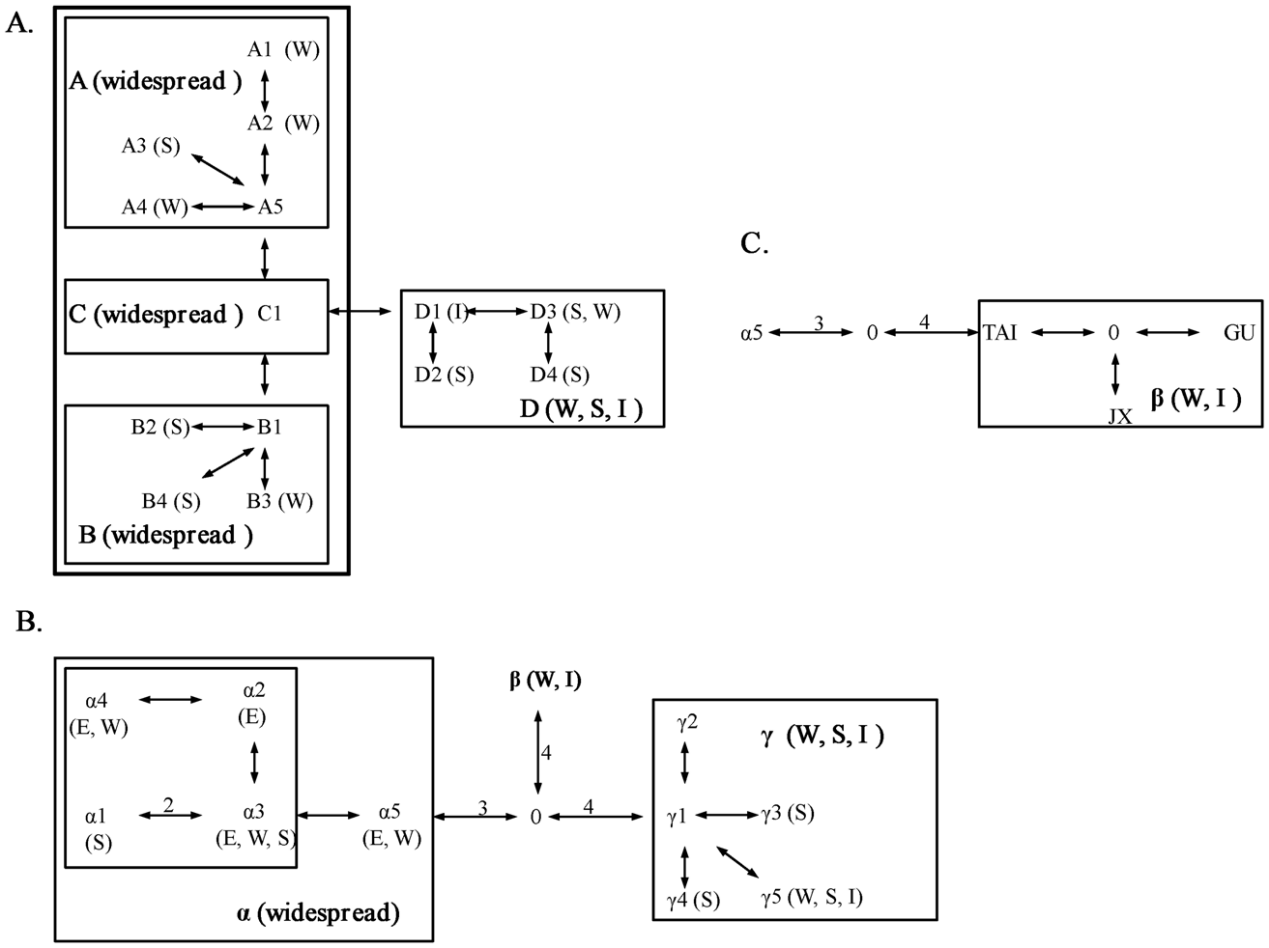
Minimum spanning network of *Pinus massoniana*. Geographical distribution of each haplotype and lineage is indicated: west (W), south (S), and island (I). A) cpDNA haplotypes; B) *AdhC2* alleles; C) lineage ß of *AdhC2* alleles.

Gene genealogy was reconstructed based on the cpDNA haplotypes of *P. massoniana*, rooted at *P. taiwanensis* individuals (accession numbers: HE972271-HE972272) (Fig. 2A). Four major lineages A, B, C, and D were identified, and a further clustering of A, B, and C was recovered. No distinct geographical subdivision was detected among the three types; all were widespread. Within cluster D, the basal haplotype D1 occurred in Taiwan only and also was dominant within the population (20/22 = 90.9%, Table 2). To understand the relative ages between haplotypes/clades, we reconstructed a minimum spanning network (Fig. 3A), based on mutational changes among cpDNA haplotypes. According to the coalescent theory, tip nodes of a network are likely to represent descendents derived from ancestral, interior nodes [25]. Gene genealogies are therefore used to infer information about migration, gene flow, hybridization, and divergence between lineages [26]. Within the minimum spanning network, clade C was nested as the interior node, independently linked to A and B. All widespread haplotypes were also nested in the network as interior nodes. Clade D was absent in populations of the eastern region; other three clades were widespread across all regions.

Likewise, the phylogeny based on sequence variation in the *AdhC2* introns was reconstructed, rooted at *P. taiwanensis* individuals (accession numbers: HE972269-HE972270) (Fig. 2B). Three major lineages (α, β, and γ) were identified, with β and γ clustered together and sister to α. Within clusters β and γ, haplotypes pmt4 and pmt6, respectively, of Taiwan were basal to others of the mainland populations. A minimum spanning network based on mutational changes among *AdhC2* alleles was reconstructed (Fig. 3B). Within β and γ, haplotypes pmt4 and pmt6 were linked to a missing node, a hypothetical ancestor (Fig. 3C).

### Microsatellite diversity within populations

Estimates of diversity varied among populations based on 11 microsatellite loci (Table 5). The average number of alleles (A) across populations ranged from 1.636 to 2.545. Yangshan population (HO) in south region had the highest number of alleles per locus. The observed heterozygosity (*H_O_*) and expected heterozygosity (*H_e_*) varied with a range of 0.09091–0.29032 and 0.18989–0.47385, respectively (Table 5). The Daiyunshan population of Fujian (DA) in south region had the highest level of genetic diversity, *H_O_* = 0.29032; whereas, Lushan and Taiwan populations had the lowest level of genetic diversity, *H_O_* = 0.09091. For all populations, significant departures from HWE were detected (P<0.05), indicating deficiency of heterozygosity.

**Table 5:** A comparison of genetic diversity at11 microsatellite loci in 8 populations of *Pinus massoniana*.

	*A*	*Ho*	*He*
**Mainland China**	4.273	0.16061	0.59492
East			
HS I	1.909	0.15978	0.29659
HS II	2.455	0.14773	0.41858
West			
JX	2.273	0.09091	0.45709
HN	2.000	0.11455	0.32505
GU	1.636	0.11515	0.24208
South			
DA	1.909	0.29032	0.32113
HO	2.545	0.16804	0.35695
**Island Taiwan**			
TAI	1.636	0.09091	0.18989
Overall	4.273	0.13949	0.59291

*A* : Observed average allele number; *Ho*: Observed heterozygosity; *He*: Expected heterozygosity.

**Figure 4:**
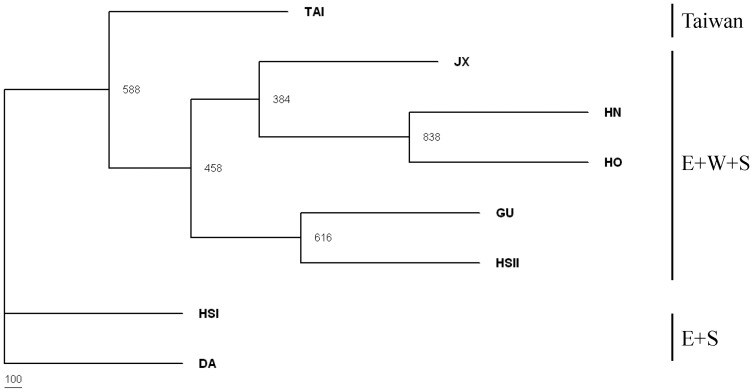
Unrooted NJ tree showing genetic relationship based on microsatellite loci among populations. Numbers at the nodes are bootstrapping values from 1000 replicates.

**Figure 5:**
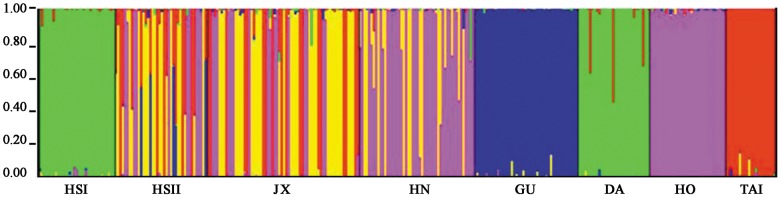
Bayesian inference of the number of clusters (K) of *Pinus massoniana* based on microsatellites. The three clusters (K = 5) were detected from STRUCTURE analysis with highest ΔK value.

An un-rooted phylogram was constructed from microsatellite frequencies using Nei's D to assess the genetic relationships (Fig. 4). Three groups were identified, HS I and DA populations clustered together, and linked to TAI and other populations in mainland China. Using the method suggested by Pritchard *et al.* [27], the inference of the number of gene pools K was not straightforward as log-likelihood values for the data conditional on K, ln P(X|K), increased progressively as K increased. In such a case it may not be possible to determine the true value of K. However, ΔK values [28] computed for all K classes indicated a strong signal for K = 5 (ΔK = 84.098567). The proportions of each individual in each population were assigned into 5 clusters. The genetic composition of populations based on STRUCTURE analysis was consistent with the neighbor-joining tree based on Nei's D (Fig. 5). A general pattern was observed that individuals in the HS I population tended to be similar to DA population, HN population tended to be similar to HO populations, and the HS II, JX, and HN populations were intermixed with other populations. The populations of GU and TAI are relatively unique, with haplotypes shared with very few individuals of other populations (Fig. 5).

### Population genetic structure and population demography

To investigate the population genetic structure of *P. massoniana*, a SAMOVA analysis (Table 6) was applied to define groups and to identify any locations of genetic uniqueness among the 8 populations. For cpDNA, an assumption with two groups (*K* = 2) displayed the greatest value of *F*_CT_ and a maximal variance (*F*_CT_ = 0.71721, 71.72%, *P*<0.05) (Table 6). The groups at *K* = 2 exactly matched geographical distribution, TAI population forming one group and those in mainland China forming the other. For nuclear DNA, the same groups were identified as cpDNA (*F*_CT_ = 0.417756, 41.78%, *P*<0.05). Microsatellite data indicated the different pattern based on SAMOVA, HS I, and DA population were grouping together, and others formed another group (F_CT_ = 0.84281, 84.28%, *P*<0.05) (Table 6).

**Table 6:** Fixation indices corresponding to groups of populations inferred by SAMOVA for *Pinus massoniana* populations tested for the cpDNA, nDNA, and microsatellites.

Marker	Group	Population groupings	Variation among group (%)	F_CT_	P
cpDNA	**2**	**(HS I, HS II, JX, HN, GU, DA, HO) (TAI)**	**71.72**	**0.71721**	**0.00000**
	3	(HS I, HS II, JX, HN, GU, DA) (HO) (TAI)	54.08	0.54078	0.03128
	4	(HS I) (HS II, JX, HN, GU, DA) (HO) (TAI)	46.84	0.46836	0.00000
	5	(HS I, HS II) (JX, HN, GU) (DA) (HO) (TAI)	45.26	0.45262	0.00000
	6	(HS I,HS II) (JX) (HN, GU) (DA) (HO) (TAI)	41.73	0.41731	0.00489
nDNA	2	**(HS I, HS II, JX, HN, GU, DA, HO) (TAI)**	**41.78**	**0.41775**	**0.00000**
	3	(HS I, HS II, JX, HN, GU) (DA, HO) (TAI)	40.70	0.40703	0.00587
	4	(HS I) (HS II) (JX, HN, GU) (DA, HO,TAI)	26.28	0.26277	0.00000
	5	(HS I) (HS II) (JX, HN, GU) (DA, HO) (TAI)	33.72	0.33721	0.00098
	6	(HS I, JX, HN) (HS II) (GU) (DA)(HO) (TAI)	33.57	0.33574	0.01760
SSR	2	**(HS I, DA) (HS II, JX, HN, GU, HO, TAI)**	**84.28**	**0.84281**	**0.00000**
	3	(HS I, DA) (HS II, JX, HN, HO, TAI) (GU)	83.24	0.83239	0.00684
	4	(HS I) (HS II, JX, HN, HO, TAI) (GU) (DA)	82.80	0.82799	0.01760
	5	(HS I) (HS II, JX, HN,TAI) (GU) (DA) (HO)	79.90	0.79897	0.00782
	6	(HS I) (HS II, JX, HN) (GU) (DA) (HO) (TAI)	79.97	0.78975	0.00000

**Figure 6:**
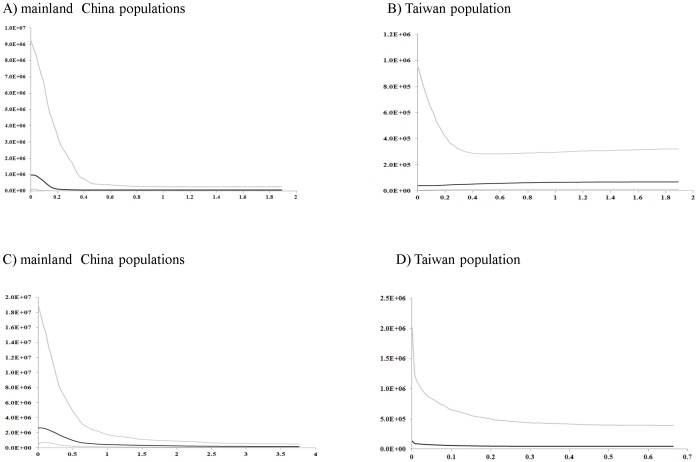
Bayesian skyline plots based on cpDNA and nDNA for the effective population size fluctuation through time. Y axis: population size × generation time; X axis: time, years before present (million years ago). Population fluctuations of A) mainland populations based on cpDNA; B) Taiwan population based on cpDNA; C) mainland populations based on nDNA; and D) Taiwan population based on nDNA are presented. Black lines represent median estimations; area between gray lines represent 95% confidence intervals).

We used mismatch distribution analyses to infer the long-term demographic history of populations. In most *P. massoniana* populations, cpDNA displayed a bimodal or multimodal mismatch distribution, except for population Huangshan I (Fig. S1). Almost all p-values are not significant, except for those for Taiwan, Guangdong, and Hunan (Fig. S1). Because the data used to produce mismatch distributions are not independent, Fu and Li's D*, Fu and Li's *F* and Tajima's *D* statistics were used for detecting departure from population equilibrium. In this study, almost all these values were nonsignificantly negative, except for Hunan (HN) and Taiwan (TAI) (Table 3). The overall analysis revealed negative values, except for Tajima's *D*. Mismatch analyses of the nuclear DNA displayed similar patterns as the cpDNA dataset, i.e., mostly bimodal or multimodal mismatch distributions (Fig. S2). Fu and Li's D*, and Fu and Li's *F* statistics were mostly nonsignificantly negative (Table 3). However, no significant deviation was detected in all populations except for Taiwan (TAI). The analyses of overall populations were significantly negative for the nuclear DNA data set, except for the Tajima's *D*. However, Tajima's *D* for the nuclear marker was mostly nonsignificantly positive within the mainland populations. Most of the analyses for detecting population demography were of negative values for both loci, but few with statistical significance. The Bayesian skyline plot were therefore used to detect the demography for the mainland and Taiwan populations of *P. massoniana* (Fig. 6). Based on the pattern of variation in cpDNA and nDNA, a long history of constant population size of *P. massoniana*, followed by subsequent demographic expansion, was uncovered. Likewise, the Taiwan population displayed a pattern with a constant population size in cpDNA, whereas a recently slow population growth in nDNA (Fig. 6).

## Discussion

### Genetic diversity and structuring in *Pinus massoniana*

*Pinus massoniana* represents a species of typical evergreen forests in central and southern China. As a dominant species at mesic and harsh habitats with a wide distribution, *P. massoniana* is expected to have high levels of genetic diversity. Nevertheless, the level of nucleotide diversity of cpDNA (0.00596) detected from 320 individuals across eight natural populations was not high. The genetic diversity approximated that of *P. hwangshanensis* (0.00605), while much lower than that of *P. luchuensis* (0.05014) and *P. taiwanensis* (0.07051) at the same locus [6]. Zhou *et al.* [29], a phylogeography study for 180 individuals of *P. massoniana*, identified 6 haplotypes with 20 polymorphic sites based on three cpDNA genes, which were relatively lower than 14 chlorotypes with 24 polymorphic sites detected in this study. The difference may simply come from the sampling difference. Besides, the level of nucleotide diversity of *AdhC2* introns (0.01142) in *P. massoniana* was lower than that of other nDNA loci, such as *lp3-3* (0.0370), *dhn1*(0.0144), and *dhn3*(0.0198), whereas higher than that of *lp3*-1(0.0095), *chcs* (0.0075), and *dhn2*(0.0074) in *P. sylvestri* [30], a result reflecting different functional constraints among genes. Furthermore, for microsatellite loci, all *P. massoniana* populations had lower allele number (1.636–2.545) and expected heterozygosity (0.18989–0.45709) (Table 5) than those in *P. radiata* (*A* = 6.730–8.470; *He* = 0.68000–0.77000) [31], and *P. contorta* (A = 6.560–11.330; *He* = 0.65200–0.75400) [32], whereas higher than those in *P. halepensis* (*A* = 1.294–1.992; *He* = 0.173–0.377) [33].

Furthermore, approximating levels of genetic diversity in another sympatrically distributed species, *P. hwangshanensis*, reflect the effects of a common geological history across the mainland, likely associated with the Quaternary glacial maxima [6], [15]. For *P. massoniana*, the existence of the higher numbers of haplotypes and nucleotide diversities at both loci in populations in the south and in Hunan (HN) of central China, and highest averaged allele numbers and observed heterozygosity (*Ho*) at micorsatellite loci in populations in the south, highlights such impacts of the climatic oscillations [34].

Range disjunctions for plant species, a pattern also occurring for *P. massoniana*, are often a consequence of past climatic changes [1]. Although recent human disturbance could also account for the demographic fragmentation, a short period for isolation is not likely to cause the dramatic loss of genetic variations or significant genetic differentiation because of the maintenance of the shared ancestral variations [6]. Seemingly, the significant geographical structuring across *P. massoniana* populations, as indicated by the SAMOVA based on cp-and nDNAs, agreed with the scenario (mainland China vs. Taiwan). In contrast, microsatellite dataset revealed a different geographical pattern likely due to the existence of numerous ancestral polymorphisms shared between populations. Such population structuring was likely a result of historical fragmentations, while recent demographic decline must have worsened the loss in genetic diversity.

### Central–marginal paradigm and multiple refugia

The genetic characteristics of populations are usually governed by the interplay of genetic drift, natural selection, gene flow, and demographic history [15]. In this study, we tested the central–marginal paradigm that predicts that populations around the distributional center tend to have higher levels of genetic variation than the peripheral ones [8]. For *P. massoniana*, although the two populations in Huangshan had the lowest levels of genetic diversity at cp- and nDNA loci likely agreeing with the paradigm prediction, populations of the southernmost populations (DA and HO) were highly polymorphic in their genetic composition of genes and microsatellite fingerprints (Fig. 1; Table 3, 5).

The mainland–island contrast in populations of *P. massoniana* provides another perfect system for testing this paradigm. Likely shaped by founder's effects, with a long isolation and located at the margins of its distributional range, island populations would have low genetic variation. Altogether, the central–marginal paradigm alone cannot explain the apportionment of the genetic variations in *P. massoniana*, even though significant genetic differentiation was detected among most populations as predicted by the paradigm. This pattern, with high diversity in marginal populations (DA and HO) and great genetic differentiation between populations (Table 3, S1, S2), somewhat contradicts a prediction for wind-pollinated species [35].

In addition, a noteworthy trend showed higher diversity in south region than in east and west regions. A cline-like distribution of genetic diversity was also found for the *P. luchuensis* complex [6], reflecting a common scenario of postglacial, northward recolonization. Such historical migrations once prevailed in most angiosperms and pines of Europe and North America, resulting in revegetation after the Quaternary glacial retreat [1]. Pleistocene glaciation also had enormous effects on the vegetation in mainland Asia, including pines, and provided a chance for plants to migrate southward. The extinction of the mainland populations in north during the glacial maximum, followed by postglacial recolonization via few migrants from refugia in the south, would have occurred. Pollen data from a long borehole in the Yangtze River delta [18] suggest that southeastern China was forested throughout the Quaternary, and *Pinus* has generally been an important component of the regional vegetation. Many Chinese subtropical *Pinus* dominated the vegetation during the late Pleistocene and Holocene with only minor population changes [19], [20]. Seemingly, the population expansion of *P. massoniana* likely occurred in the past geological history, as also indicated by the negative Tajima's *D* and Fu's *Fs* statistics in cp- and nDNAs (Table 3). That is, the population decline (i.e., severe bottleneck) only occurred recently. As glaciers displaced pine distributions profoundly at high latitudes, most plant species/populations at middle latitudes, where no ice sheets covered the land mass, underwent frequent expansion and contraction from global cooling [36]. This process apparently resulted in low levels of genetic diversity in the Huangshan populations (east region) of *P. massoniana* and *P. hwangshanensis* [6].

Furthermore, Zhou *et al.* [29] suggested multiple glacial refugia that fostered populations with high genetic diversity had existed in southern populations of *P. massoniana* and *P. hwangshanensis*. In this study, a scenario of multiple glacial refugia in south region was also recovered. The multiple colonizing events were likely to contribute to the high heterogeneity of the genetic composition of populations in the southern region during the largest Quaternary glacial ages; populations DA and HO had all cpDNA lineages, and most nuclear DNA types except for lineage β (Table 2, 4). The great number of haplotypes and private alleles at cp- and nDNA loci and the high genetic diversities detected in microsatellite fingerprinting suggested that these populations likely represent some glacial refugia during the largest Quaternary glacial ages in Asia. Such southward colonization into glacial refugia agrees with a migratory pattern with colonists recruited from a random sample of previously existing populations during the glacial maxima, apparently resulted in the high levels of genetic diversity in these marginal populations in the south China.

Likewise, Taiwan, as a subtropical continental island, also provided a refugium for the southward migrants [15]. The expansion of glaciers caused a drop in sea levels of the South China Sea up of 120 m, leading to the emergence of a land bridge between Taiwan and the mainland [37]. This inter-land corridor provided opportunities for the southward migration of the refuge species into the island refugia, while subsequent harsh climates kept island populations from migrating back to the mainland [6]. Like many other plants in Taiwan and the Ryukyus, high levels of genetic diversity were detected in the single population of *P. massoniana*, as indicated by the existence of lineages B and D (cpDNA), and lineages β and γ (nuclear DNA intron).

Here, the inconsistency between cpDNA and nDNA phylogenies may have stemmed from different evolutionary rates, selection, recombination, or/and DNA linkage [38]. The genetic distinctness of the Taiwan population further suggested geographical barriers for gene flow between the Taiwan Strait ever since the Pleistocene separation. Following the Pleistocene separation, it is highly possible that the populations of *P. massoniana* within mainland and island were affected by population expansion after the Last Glacial Maximum (LGM), approximately 18000–20000 years ago (Fig. 6). A SAMOVA revealed significant geographical structuring (mainland China vs. Taiwan) across the distributional range at cpDNA and nDNA loci (Table 6). Dominance of the private chlorotype D1 and the absence of chlorotypes A and C differentiated the Taiwan population from the mainland populations (Table 2). Pairwise Fst values among populations in all genes and microsatellites also revealed such geographical structuring (Table S1, S2).

Interestingly, the genetic composition suggests that populations of Huangshan (eastern region) were likely to be intermixed between populations of the south and west (Fig. 1). Furthermore, the cpDNA composition was dominated by lineage B, and nuclear DNA was mostly composed of lineage α, altogether reflecting phylogenies associated with south and west regions, and agreeing with the nature of postglacial recolonization (Fig. 1). Microsatellite fingerprinting also revealed such intermixed genetic composition of genotypes of south and west regions (Fig. 5). The two populations in Huangshan (east region) are geographically close to each other, whereas were genetically differentiated as indicated by the STRUCTURE analysis. The HS I populatuion was close to DA population in the genetic composition, while HS II population is admixed with elements of JX, HN, and HO populations, seemingly suggesting frequent gene flow between populations. Nevertheless, the pattern of genetic polymorphisms shared by geographically distant populations, here HS I vs. DA, as well as HS II vs. JX, HN, and HO populations, contradicts a pattern of “isolation by distance”. Recent fragmentations of a panmictic population could lead to random apportionment of genetic polymorphisms across fragments. That is, in addition to recurrent gene flow, maintenance of ancestral polymorphism via stochatical genetic drift can possibly result in high affinity between distant populations. This phenomenon has been well documented in *Drosophila* [39], *Quercus* [40], [41], and *Cycas* [42].

### Ancestral population size, and demographic expansion

As a dominant species distributed in central and southern China, *P. massoniana* is expected to have had a large ancestral population. In this study, the BEAST program was used to estimate fluctuations of the ancestral effective population size in mainland populations and in the island population based on Bayesian skyline plots. Larger effective population sizes in the mainland than in Taiwan were detected with cp- and nDNAs. Based on both loci, a long history of constant effective population size of *P. massoniana* was detected across mainland populations, followed by subsequent demographic expansion. Likewise, Taiwan population of *P. massoniana* almost remained nearly constant before a recent population expansion in the nuclear DNA, whereas, a constant and stable population size was detected in cpDNA. Following the above, mainland and Taiwan populations did not undergo large fluctuations in population size until its recent population expansions (Fig. 6). A similar scenario of population expansion based on Bayesian skyline plots was also detected in *Cyacs revoluta* of Japan and China, and *C. taitungensis* in Taiwan [42].

Ecologically, *P. massoniana* forest is a typical secondary vegetation type on the denuded hill slopes of subtropical Southeast China [43]. Apparently, past fragmentation before *P. massoniana* became dominant in the secondary forests 1100 years ago in southern China may have triggered the genetic differentiation between populations/geographical regions. A demographic expansion, which was well documented in most plants of East Asia [15], occurred in the pine populations, as revealed by the negative values in Tajima's *D* and Fu's *Fs* statistics (Table 3), as well as the deviation of mismatch distribution analyses, though nonsignificant (Fig. S1, S2). Although population fragments remained isolated from each other, the microsatellite fingerprinting indicated that the populations were mediated by some degree of gene flow.

We investigated the phylogeography and population structuring of *Pinus massoniana* based on the genetic variation of cp-, nDNAs and microsatellites. A history of postglacial recolonization of the populations and multiple refugia, subsequently affected by demographic expansion after glaciations, was inferred. High levels of genetic diversity of the marginal populations in the south region of mainland suggested that the climatic changes might have low impacts on these populations at low latitudes. These results provide a phylogeographical hypothesis for further testing. A large number of low-copy nuclear genes would be required for examining the genomic divergence within *P. massoniana*.

## Materials and Methods

### Ethics Statement

This study was conducted in accordance with the laws of China and Taiwan. The locations of field studies are not privately-owned or protected areas, and are not involved with endangered or protected species. No permits were required for this study.

### Population sampling and DNA extraction

Eight populations of *Pinus massoniana* throughout its whole geographical range were surveyed (Fig. 1). Although this species has a wide range in mainland China, natural populations are patch-like and scattered across southern and central China. According to the geographical distribution of the natural populations, HS I, HS II, DA, and HO populations were recognized as the peripheral populations, while others as the central populations in mainland China. Sampling from plantation populations or between short distances was avoided (>50 m); only native populations were included for analyses. In total, 320 individuals were collected (Table 1). Young, healthy leaves were sampled and dried in silica gel until DNA extraction. Total genomic DNA was extracted from leaves using a CTAB methodology [44].

### Organelle and nuclear DNA analyses

Variation within and between populations was analyzed for the organelle DNAs and *AdhC2* introns 4 to 8 in nuclear DNA. PCR amplification was carried out in a 100-µL reaction. The reaction was optimized and programmed on a MJ Thermal Cycler (PTC 100) for one cycle of denaturation at 95°C for 4 min; 30 cycles of 45 s denaturation at 94°C, 75 s annealing at 52°C, and 90 s extension at 72°C; then 10 min extension at 72°C. Template DNA was denatured with reaction buffer, MgCl_2_, NP-40 and ddH_2_O for 4 min (first cycle), and cooled on ice immediately. Primers, dNTP, and *Taq* polymerase (Takara Bio Inc., Shiga, Japan) were added to the ice-cold mixture. Primers were synthesized for amplifying the *atp*B-*rbc*L intergenic spacer (*atp*B-1: 5′-ACATCKARTACKGGACC AATAA-3′ and *rbc*L-1: 5′-AACACCAGCTTTRAATCCAA- 3′) [45], and *AdhC2* introns (Pinus-*AdhC2* exon4-F5′-TAC ATC GAC TTT CAG CGA GTA C-3′ and Pinus-*AdhC2* exon 9-R 5′-TATG ATA GAA GCC TTA CCT TAG C-3′) [46].

PCR products were electrophoresed on agarose gels, and the desired fragments were excised from the gel and purified. For the *AdhC2* introns, purified DNAs were sequenced directly or ligated to a pGEM-T easy vector (Promega, Madison, WI, USA) if the heterozygote was directly sequenced. Five randomly selected clones were purified to obtain DNA plasmids using the Plasmid DNA Extraction Miniprep System (Viogene-BioTek Co., Taipei, Taiwan). Eluted PCR products and DNA plasmids were sequenced directly in both directions by standard methods of the *Taq* dye deoxy terminator cycle sequencing kit (Perkin-Elmer, Wellesley, MA, USA). Products of the cycle sequencing reactions were run on an ABI 377XL automated sequencer (Applied Biosystem, Foster City, CA, USA).

### Microsatellite genotyping

Eleven variable microsatellite regions [47] were amplified following the method described in the below. PCR amplification was carried out in a 50-µL reaction. The reaction was optimized and programmed on a MJ Thermal Cycler (PTC 100) for one cycle of denaturation at 95°C for 4 min; 30 cycles of 30 s denaturation at 94°C, 1 min at proper annealing temperatures [48], and 60 min extension at 72°C; then 10 min extension at 72°C. The PCR reaction mixture contained 50 mM Tris-HCl (pH = 8.0), 500 mg/ml KCl, 1.5 mM MgCl_2_, 200 µM dNTP and 0.4 µM (each) primer; the forward primers [48] were labeled with fluorescent dye, 6-FAM, TAMRA or HEX (Applied Biosystems), 20 ng of DNA template, and 0.6 U Taq polymerase. The PCR products were separated on an ABI 3100 automated sequencer using a 50-cm capillary, polymerPOP-6 and ROX 500 (both Applied Biosystems) as an internal standard. Fragment sizes were assessed using GENEMAPPER software version 3.7 (Applied Biosystems). Allele size was indicated twice manually to reduce scoring error.

### Phylogenetic and phylogeographical analyses of DNA sequences

Nucleotide sequences were aligned with the program BlastAlign [48]. After the alignment, the indels were coded as binary characters. Maximum-likelihood (ML) analyses were performed using PHYLIP v. 3.67 [49] on each locus. The general time reversible HKY model was determined by Modeltest v3.6 [50] to be the most suitable model and was used for the subsequent nucleotide analyses. Confidence in the reconstructed lineages was tested by bootstrapping [51] with 1000 replicates using unweighted characters.

A network analysis complementary to conventional cladistics provides the power to discriminate between phylogeographical patterns that result from restricted gene flow and those that arise from historical events at the population level [52]. The number of mutations between DNA sequence haplotypes in pairwise comparisons were estimated from the data using the Molecular Evolutionary Genetics Analysis program (MEGA, version 5.0) [53], then used to construct a minimum spanning network [6]. Networks were produced with the program MINSPNET [54].

### Population genetic analysis

Summary statistics such as the number of haplotypes (h) and minimum number of recombinations (Rm) were determined. The level of genetic diversity within populations was quantified by measures of nucleotide diversity θ [55] using DnaSP Version 5.1 [56]. To make inferences about demographic changes of *P. massoniana*, we employed both mismatch distributions and statistical tests of neutrality. We calculated Tajima's *D* [57], and Fu and Li's D* statistic [58] in the noncoding DNA fragments as indicators of demographic expansion in DnaSP.

SAMOVA (spatial analysis of molecular variance) was applied to identify groups of geographically adjacent populations that were maximally differentiated based on sequence data [59]. We performed the analyses based on 100 simulated annealing steps and examined maximum indicators of differentiation (*F*_CT_ values) when the program was assigned to identify *K* = 2–6 partitions of populations.

We also investigated historical demographics of populations by plotting mismatch distributions [60] and comparing them to Poisson distributions. The parameters of demographic expansion were estimated using the methods of Schneider and Excoffier [61].

### Microsatellite fingerprinting analysis

For microsatellite loci, genetic diversity within populations was assessed by calculating allele number (A), observed (*Ho*), and expected (*He*) heterozygosities by using the Arlequin program version 3.1 [62]. The program is also performed to calculate population differentiation level (Fst), to analyze molecular variance (AMOVA), and to test Hardy-Weinberg equilibrium (HWE). SAMOVA (spatial analysis of molecular variance) was performed to identify groups of geographically adjacent populations based on microsatellite data [59]. A NJ tree with bootstrapping values from 1000 replicates was constructed in comparison for eight populations using the PHYLIP Package v. 3.67 [49]. STRUCTURE version 2.1 [27], [63] applies a Bayesian method to infer the number of clusters (K) without using prior information of individual sampling locations. This program distributes individuals among K clusters based on their allelic frequencies and estimates the posterior probability of the data given each particular K. STRUCTURE was run for K = 1 to K = 9 clusters. Each run was pursued for 1 000,000 MCMC interactions, with an initial burn-in of 100,000 and an ancestry model that allowed for admixture, with the same alpha for all populations. To assess stability, 10 independent simulations were run for each K. The final posterior probability of K, Pr(X|K), was computed, as suggested by Pritchard *et al.* [27], using the runs with highest probability for each K. However, as indicated in the STRUCTURE documentation and Evanno *et al.* [28], Pr(X|K) usually plateaus or increases slightly after the ‘right’ K is reached. Thus, following Evanno *et al.* [28], ΔK, where the modal value of the distribution is located at the real K, was calculated.

## Supporting Information

Figure S1 **Mismatch distribution of cpDNA haplotypes based on pairwise sequence differences against the frequencies of occurrence for seven mainland China and one Taiwan populations of** ***Pinus massoniana*** **.** The number of pairwise nucleotide differences between haplotypes is represented on the X axis; their frequencies are represented on the Y axis.
(TIF)

Figure S2 **Mismatch distribution of nDNA haplotypes based on pairwise sequence differences against the frequencies of occurrence for seven populations of** ***Pinus massoniana*** **from mainland China and one from Taiwan.** The number of pairwise nucleotide differences between haplotypes is represented on the X axis; their frequencies are represented on the Y axis.
(TIF)

Table S1 **Pairwise F_ST_ among populations deduced from sequences of cpDNA (above the diagonal) and nDNA (below the diagonal) for** ***Pinus massoniana*** **.**
(DOC)

Table S2 **Pairwise F_ST_ among populations deduced from microsatellites dataset for** ***Pinus massoniana*** **.**
(DOC)
