# Supplementary figures and images for: Inferring Multiple Refugia and Phylogeographical Patterns in Pinus massoniana Based on Nucleotide Sequence Variation and DNA Fingerprinting
Source: PLoS One. 2012 Aug 29;7(8):e43717. doi: 10.1371/journal.pone.0043717 (PMC3430689; doi:10.1371/journal.pone.0043717)

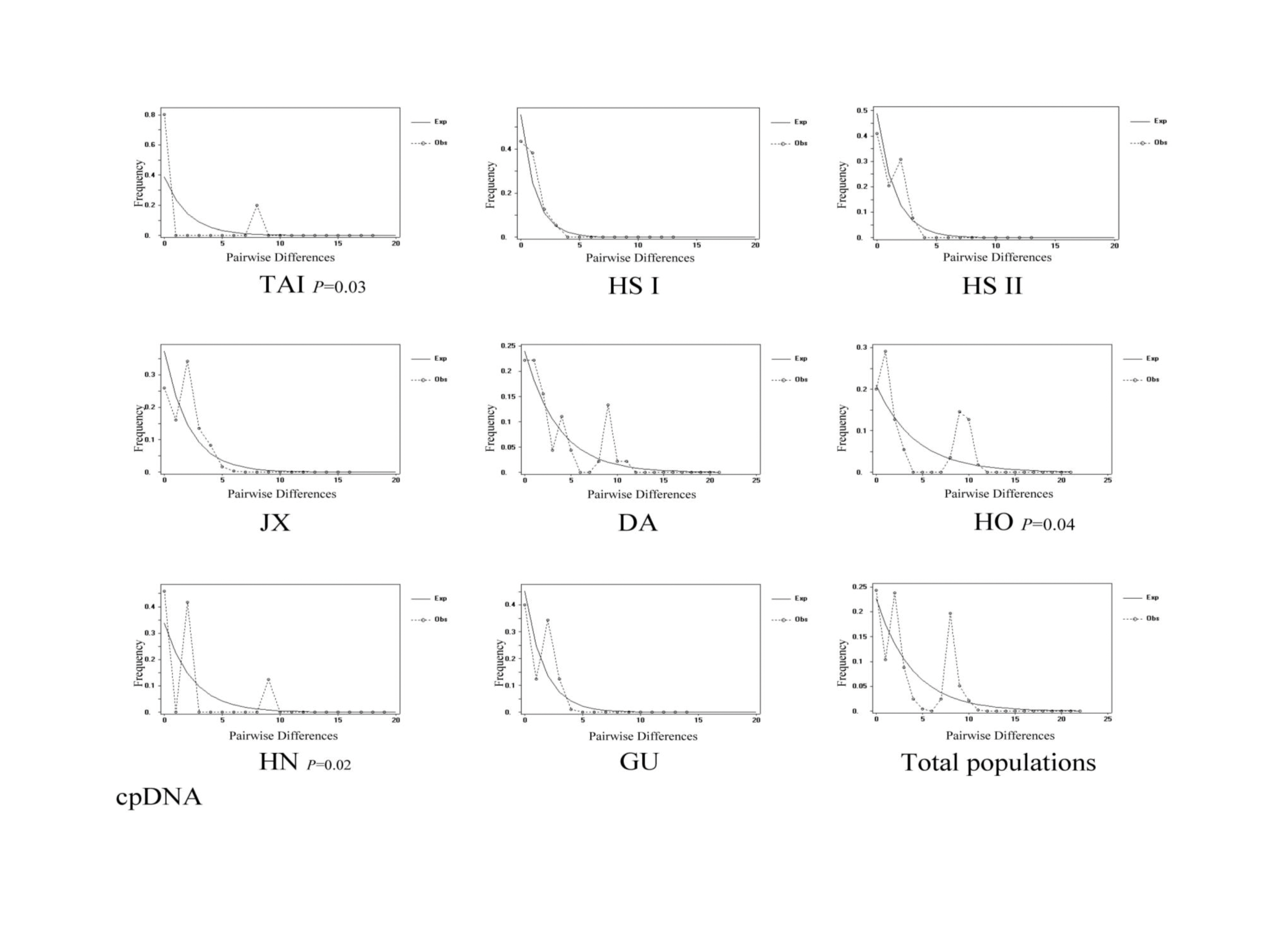

Supplement: Figure S1 — Mismatch distribution of cpDNA haplotypes based on pairwise sequence differences against the frequencies of occurrence for seven mainland China and one Taiwan populations of Pinus massoniana . The number of pairwise nucleotide differences between haplotypes is represented on the X axis; their frequencies are represented on the Y axis. (TIF) [file pone.0043717.s001.tif]

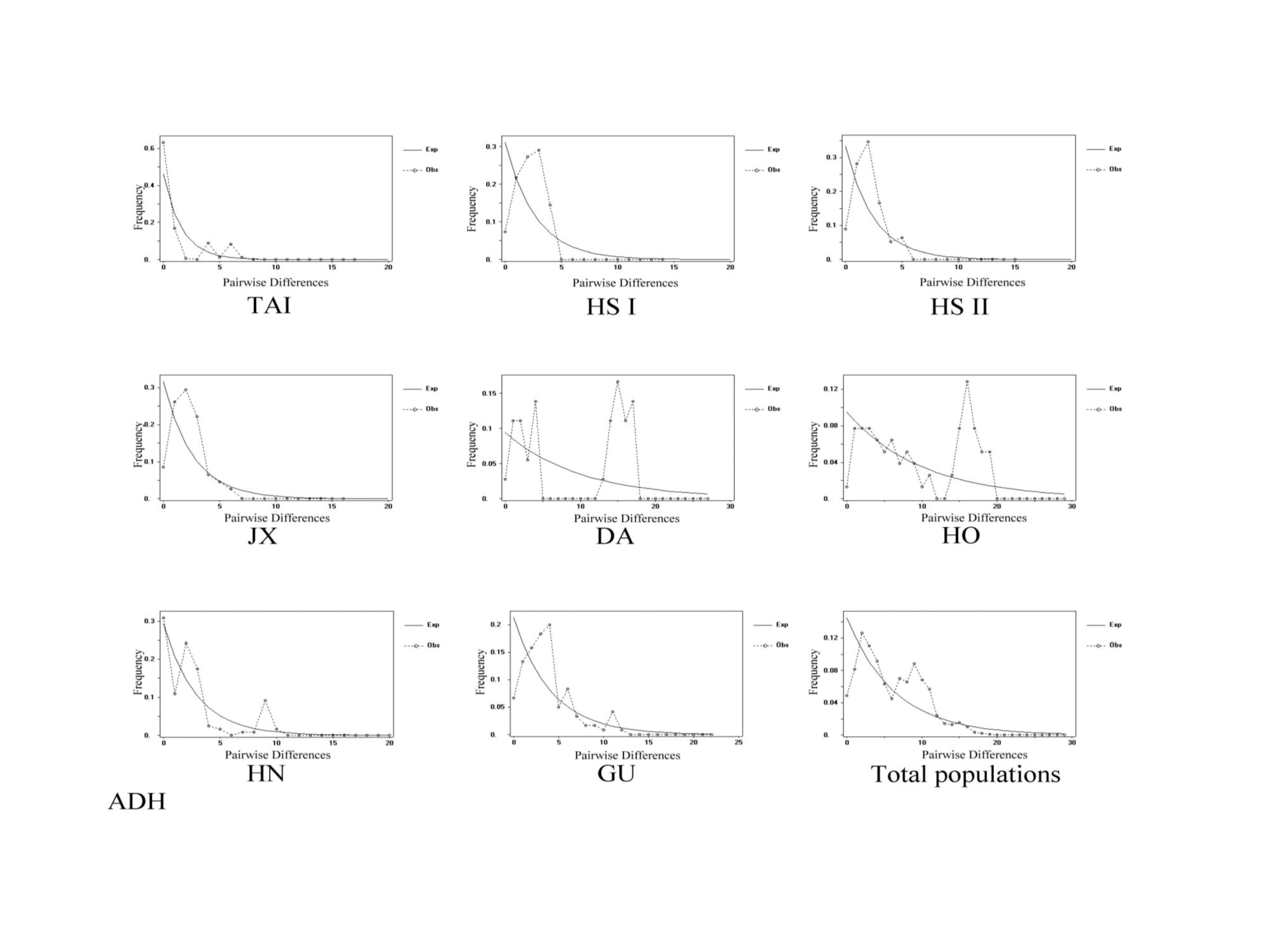

Supplement: Figure S2 — Mismatch distribution of nDNA haplotypes based on pairwise sequence differences against the frequencies of occurrence for seven populations of Pinus massoniana from mainland China and one from Taiwan. The number of pairwise nucleotide differences between haplotypes is represented on the X axis; their frequencies are represented on the Y axis. (TIF) [file pone.0043717.s002.tif]
