# Supplementary material for: Inferring Multiple Refugia and Phylogeographical Patterns in Pinus massoniana Based on Nucleotide Sequence Variation and DNA Fingerprinting
Source: PLoS One. 2012 Aug 29;7(8):e43717. doi: 10.1371/journal.pone.0043717 (PMC3430689; doi:10.1371/journal.pone.0043717)
Supplement: Table S1 — Pairwise FST among populations deduced from sequences of cpDNA (above the diagonal) and nDNA (below the diagonal) for Pinus massoniana . (DOC) [file pone.0043717.s003.doc]

Table S1. Pairwise F_ST_ among populations deduced from sequences of cpDNA (above the diagonal) and nDNA (below the diagonal) for *Pinus massoniana.*

|  | **mainland** | **East** |  |  | **West** |  |  |  | **South** |  |  | **Taiwan** |  |
| --- | --- | --- | --- | --- | --- | --- | --- | --- | --- | --- | --- | --- | --- |
|  | **China** |  | HS I | HS II |  | JX | HN | GU |  | DA | HO |  | TAI |
| **mainland**  **China** |  |  |  |  |  |  |  |  |  |  |  | 0.76060 |  |
| **East** |  |  |  |  | 0.30389 |  |  |  | 0.07162 |  |  | 0.84464 |  |
| HS I |  |  |  | 0.03242 |  | 0.37370 | 0.37240 | 0.45303 |  | 0.06884 | 0.10016 |  | 0.85630 |
| HS II |  |  | 0.29624 |  |  | 0.21618 | 0.22870 | 0.28441 |  | 0.03968 | 0.09274 |  | 0.83668 |
| **West** |  | 0.10732 |  |  |  |  |  |  | 0.18019 |  |  | 0.80303 |  |
| JX |  |  | 0.05874 | 0.25006 |  |  | 0.02126 | 0.01761 |  | 0.15354 | 0.20974 |  | 0.81201 |
| HN |  |  | 0.05089 | 0.34888 |  | 0.10503 |  | 0.00066 |  | 0.13493 | 0.17642 |  | 0.77094 |
| GU |  |  | 0.06697 | 0.29616 |  | 0.06779 | 0.06734 |  |  | 0.18577 | 0.23761 |  | 0.82711 |
| **South** |  | 0.28889 |  |  | 0.03120 |  |  |  |  |  |  | 0.63501 |  |
| DA |  |  | 0.29848 | 0.30290 |  | 0.29585 | 0.29906 | 0.22447 |  |  | 0.00559 |  | 0.66525 |
| HO |  |  | 0.32256 | 0.34514 |  | 0.32292 | 0.31053 | 0.25207 |  | 0.01396 |  |  | 0.60633 |
| **Taiwan** | 0.55986 | 0.73936 |  |  | 0.68994 |  |  |  | 0.28151 |  |  |  |  |
| TAI |  |  | 0.74745 | 0.77796 |  | 0.25006 | 0.70581 | 0.64877 |  | 0.34455 | 0.22571 |  |  |
