# Supplementary material for: Inferring Multiple Refugia and Phylogeographical Patterns in Pinus massoniana Based on Nucleotide Sequence Variation and DNA Fingerprinting
Source: PLoS One. 2012 Aug 29;7(8):e43717. doi: 10.1371/journal.pone.0043717 (PMC3430689; doi:10.1371/journal.pone.0043717)
Supplement: Table S2 — Pairwise FST among populations deduced from microsatellites dataset for Pinus massoniana . (DOC) [file pone.0043717.s004.doc]

Table S2. Pairwise F_ST_ among populations deduced from microsatellites dataset for *Pinus massoniana.*

|  | **mainland** | **East** |  |  | **West** |  |  |  | **South** |  |  | **Taiwan** |  |
| --- | --- | --- | --- | --- | --- | --- | --- | --- | --- | --- | --- | --- | --- |
|  | **China** |  | HS I | HS II |  | JX | HN | GU |  | DA | HO |  | TAI |
| **mainland**  **China** |  |  |  |  |  |  |  |  |  |  |  |  |  |
| **East** |  |  |  |  |  |  |  |  |  |  |  |  |  |
| HS I |  |  |  |  |  |  |  |  |  |  |  |  |  |
| HS II |  |  | 0.48835 |  |  |  |  |  |  |  |  |  |  |
| **West** |  | 0.17894 |  |  |  |  |  |  |  |  |  |  |  |
| JX |  |  | 0.43053 | 0.22807 |  |  |  |  |  |  |  |  |  |
| HN |  |  | 0.49814 | 0.37398 |  | 0.27181 |  |  |  |  |  |  |  |
| GU |  |  | 0.66831 | 0.42109 |  | 0.44975 | 0.59872 |  |  |  |  |  |  |
| **South** |  | 0.13597 |  |  | 0.17576 |  |  |  |  |  |  |  |  |
| DA |  |  | 0.34931 | 0.40115 |  | 0.33580 | 0.54436 | 0.59130 |  |  |  |  |  |
| HO |  |  | 0.49724 | 0.40471 |  | 0.30669 | 0.21138 | 0.58599 |  | 0.50350 |  |  |  |
| **Taiwan** | 0.31862 | 0.37282 |  |  | 0.42653 |  |  |  | 0.36889 |  |  |  |  |
| TAI |  |  | 0.60416 | 0.49977 |  | 0.40547 | 0.62656 | 0.64857 |  | 0.55432 | 0.64244 |  |  |
